# Digestive enzyme replacement in very preterm infants: a prospective observational study

**DOI:** 10.1038/s41430-026-01792-5

**Published:** 2026-08-04

**Authors:** Sina Keidel, Sven Wellmann, Holger Michel, Christoph Bührer

**Affiliations:** 1 Department of Neonatology, Charité Universitätsmedizin Berlin, Berlin, Germany; 2 Departmennt of Neonatology, University Children’s Hospital Regensburg (KUNO) at the Hospital St. Hedwig of the Order of St. John, University of Regensburg, Regensburg, Germany

**Keywords:** Paediatrics, Malnutrition

## Abstract

**Objectives:**

Very preterm infants may display poor postnatal growth despite receiving recommended enteral intakes. This has been linked to transient exocrine pancreatic insufficiency, indicated by low fecal pancreatic elastase-1 (FPE-1) concentrations. We aimed to determine whether growth velocity may be improved by exogenous digestive enzyme replacement.

**Methods:**

In this bicentric prospective observational study, changes in growth velocity were assessed in very preterm infants (<32 weeks gestational age, <1500 g birth weight) with low FPE-1 concentrations (<200 µg/g). Infants received 350 U lipase from *Rhizopus oryzae* and 5.4 U protease from *Aspergillus oryzae* with every meal via gavage; the dosage was doubled once infants reached 1000 g. Average daily weight gain relative to body weight was compared during the 14 days preceding initiation of digestive enzyme replacement and during the first 14 days thereafter.

**Results:**

In 71 infants (median [interquartile range] birth weight 700 [570–980] g, gestational age 25.4 [24.4–28.4] weeks) born between March 1, 2022, and December 31, 2023, daily weight gain increased from 14.7 [7.3–19.7] to 19.5 [15.7–24.0] g/kg/d (*p* < 0.001), relative daily weight gain per kg protein fed from 4.0 [2.0–5.9] to 5.4 [4.4–6.5] g/kg (*p* < 0.001), and relative daily weight gain per kcal fed from 0.11 [0.06–0.16] to 0.15 [0.13–0.19] g/kcal/d (*p* < 0.001). Effects were non-significant in infants fed raw milk but marked in those who received pasteurized mother’s own milk or donor milk.

**Conclusion:**

Administration of exogenous digestive enzymes was associated with increased weight gain in very preterm infants with transient exocrine pancreatic insufficiency.

## Introduction

While nutritional support of very preterm infants is aimed at achieving neonatal growth that matches fetal growth [1], many of these infants fail to grow along intrauterine trajectories [2, 3]. Adequate growth often remains an unachieved goal despite provision of increased amounts of protein and calories following international guidelines, such as those issued by the European Society of Pediatric Gastroenterology Hepatology and Nutrition (ESPGHAN) [1]. Early enteral feeding has been found to be important for a various health outcomes of preterm infants [4] while postnatal growth faltering has been associated to retinopathy of prematurity [5–7] and long-term neurodevelopmental impairment [8–13]. It is therefore important to aim for better growth rates in very preterm infants.

Postnatal growth faltering in very preterm infants has been linked to transient exocrine pancreatic insufficiency, which limits the digestion of proteins and fats, resulting in poor growth [14–16]. Fortified human milk or formula may not be fully digested by immature infants who have clinical and laboratory evidence of transient exocrine insufficiency [17]. At 4 weeks of age, a considerable proportion of infants with a gestational age below 28 weeks may display fecal pancreatic elastase-1 (FPE­1) concentrations below 200 µg/g, indicative of exocrine pancreatic insufficiency [17–19]. The proteolytic enzyme pancreatic elastase-1 is released alongside other digestive enzymes by the pancreas into the gut lumen and remains stable during intestinal passage [20]. Determination of FPE-1 in stool by enzyme-linked immunosorbent assays is a reliable test for quantifying the function of the exocrine pancreas also in premature infants from two weeks of age [18, 21]. FPE-1 concentrations gradually reach values considered normal (≥200 µg/g) as the pancreas matures and the infant grows, commensurate with the degree of immaturity [18, 19] The reference range of less than 200 μg/g feces can be applied to both adults and children for the diagnosis of exocrine pancreatic insufficiency, including newborns with cystic fibrosis [18, 20, 22, 23]. Sensitivity is high while specificity is limited [24].

Previous retrospective analyses suggested that enteral administration of either porcine pancreatic enzymes [25, 26] or microbial digestive enzymes derived from *Rhizopus oryzae* and *Aspergillus oryzae* [15] is associated with improved growth in such infants. In contrast to enzymes retrieved from porcine pancreas tissue, the microbial enzymes do not require acid-resistant coating and may be given through small-bore gavages. Furthermore, a mixture of these microbial enzymes is licensed to treat patients with exocrine pancreatic insufficiency in Germany without age restriction [27]. No safety issues have emerged in the studies reporting on treating preterm infants with digestive enzymes of porcine or microbial origin [15, 25, 26] but the efficacy of the digestive enzymes has never been evaluated in a prospective fashion.

This prospective observational study assessed weight gain in very preterm infants on full enteral feeds according to current ESPGHAN recommendations before and after starting administration of exogenous digestive enzyme replacement.

## Methods

This study was designed as a prospective before-after observational study conducted at two university hospitals in Berlin and Regensburg, Germany. Inclusion criteria were gestational age below 32 weeks and birth weight below 1500 g, decreasing weight percentiles while receiving full enteral nutrition in line with ESPGHAN recommendations, FPE-1 levels below 200 μg/g (determined by enzyme-linked immunosorbent assay), subsequent treatment with exogenous digestive enzyme replacement, and written informed parental consent. Infants with necrotizing enterocolitis, focal intestinal perforation, or abdominal surgery for other reasons were excluded.

Both hospitals followed similar standard operating procedures for enteral nutrition based on ESPGHAN recommendations. Milk feeding was started on the first day of life, preferably using fresh colostrum or pasteurized donor milk. Parenteral nutrition was started in parallel and gradually reduced as enteral intake increased. Milk of cytomegalovirus-positive mothers was pasteurized until infants reached 32 weeks postmenstrual age to prevent lactogenic postnatal cytomegalovirus infections [28–30]. Breast milk or donor milk was fortified with powdered commercial fortifiers containing partially hydrolyzed protein (Aptamil FMS, Danone, Frankfurt, Germany, or FM85, Nestlé, Frankfurt, Germany) once a daily volume of 100 ml was reached. Fortification continued until the infants reached a postmenstrual age of 34 weeks and a body weight of 1800 g. Target nutritional values were 150–180 ml/kg/day of fluids, 110–135 kcal/kg/day, and 4.0–4.5 g/kg/day of protein (based on estimated values without the use of a milk analyzer).

In both hospitals, exogenous digestive enzyme replacement was done using the same commercially available mixture of acid-resistant digestive enzymes derived from rice fungus cultures containing lipase (from *Rhizopus oryzae*), protease (from *Aspergillus oryzae*) and amylase (Nortase, Repha, Langenhagen, Germany). Dosages were based on previous reports using digestive enzymes of porcine or microbial origin in preterm infants [15, 25, 26]. In Berlin, capsules containing 7000 units lipase, 54 units protease and 700 units of amylase, were freshly dissolved in 4 ml of water, yielding a concentration of 1750 units/ml lipase and 13.5 units/ml protease. Dosages started at 0.2 ml per meal and increased to 0.4 ml for infants weighing over 1000 g. In Regensburg, the Nortase capsules were dissolved to a final concentration of 2500 units/ml lipase and 19.25 units/ml protease, with 0.4 ml given every other feed. Exogenous digestive enzyme replacement was restricted to infants with gavage feeding and always discontinued when the feeding tube was no longer required.

The primary outcome was average daily weight gain relative to body weight, assessed in infants on full enteral feeds up to 14 d prior to institution of digestive enzyme replacement and during the first 14 d with digestive enzyme replacement. Daily weight gain relative to body weight was calculated by dividing the weight differences by the arithmetic weight means and the number of days elapsed between measurements. Secondary endpoints were weight gain relative to calorie and protein intake, as well as changes in weight for gestational age percentile according to established growth charts [31, 32] calculated by Ped(z) Kinderarzt Rechner [33].

Statistical analysis was performed using SPSS 30.0 (IBM, Armonk, New York, USA) and Epi Info (Center for Disease Control, Atlanta, Georgia, USA). Pairwise comparisons of continuous variables were made using the Wilcoxon signed-rank test, and categorical data of groups were compared by Fisher exact test. Two-sided *p* values < 0.05 were assumed to indicate significance.

The study received ethical approval from the respective ethics committees (Berlin: EA2/004/22; Regensburg: 23-3236-104), and informed consent was obtained from the parents of all participants. The study was not registered as it was an observational study without interventional assignment.

## Results

### Patient and clinical characteristics

**Fig. 1:**
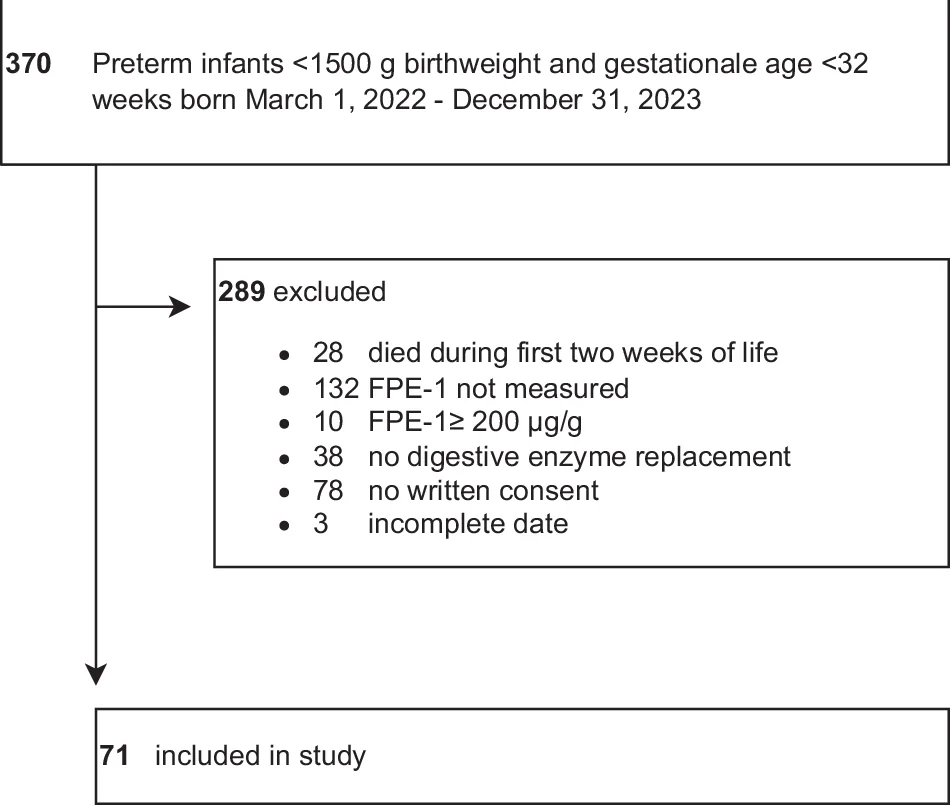
Flow diagram of patients recruited or not recruited to the study.

A total of 370 preterm infants below 1500 g birth weight and below 32 weeks gestational age were born between March 1, 2022 and December 31, 2023 and admitted to the neonatal intensive care units involved in the study, of whom 71 were recruited (Fig. 1).

Patient characteristics are given in Table 1.

**Table 1:** **a** Patient characteristics, baseline and clinical outcome; **b** Patient characteristics at initation of digestive enzyme replacement.

a	
Patient characteristics	
	*n* = 71
Sex	
Female	38 (53%)
Male	33 (47%)
Gestational age (weeks.days)	25.3 (24.3–28.3)
Gestationale age <28 weeks	51 (72%)
Birth weight (g)	700 (570–905)
<1000 g	54 (76%)
Multiple pregnancy	
Singleton	49 (69%)
Twin	19 (27%)
Triplet	2 (3%)
Intraventricular hemorrhage	
0°	52 (73%)
1°	10 (14%)
2°	4 (6%)
3°	5 (7%)
Patent ductus arteriosus	
No therapy	46 (65%)
Ibuprofen	20 (28%)
interventional or operative procedure	4 (6%)
Bronchopulmonary dysplasia	
None	27 (38.0%)
O_2_ supplementation at 28 days of life	23 (32.4%)
….at 36 weeks’ postmenstrual age	7 (9.9%)
…. at discharge	13 (18.3%)
Retinopathy of prematurity	
0°	35 (49%)
1°	6 (9%)
2°	19 (27%)
3°	10 (14%)

b	
Patient characteristics	
	*n* = 71
Fecal pancreatic elastase-1 (µg/g)	51 (21–89)
FPE -1 <100 µg/g	56 (79 %)
Digestive enzyme replacement	
Chronological age at start (days)	25 (21–29)
Postmenstrual age (weeks.days)	29.5 (28.2–31.6)
Weight at start (g)	1000 (790–1290)
Weight percentile at start	16 (7–28)
Type of feeding	
Mother’s own milk, raw	26
Mother’s own milk, pasteurized	29
Donor milk (pasteurized)	9
Mixed	7
Duration of digestive enzyme replacement (d)	40 (24–58)

Data are presented as numbers (percentage) of subjects or median [interquartile range].

### Nutrition before and after start of exogenous digestive enzyme replacement

Calculated intake of fluids, energy and proteins was rather lower after, as compared to before initiation of exogenous digestive enzyme replacement (Table 2).

**Table 2:** Intake of fluids, energy and proteins (median [interquartile range]) before and after initiation of exogenous digestive enzyme replacement.

	Before initiation	After initiation	*P*-value
Fluids (ml/kg/d)	167 (155–178)	161 (152–170)	0.001
Energy (kcal/kg/d)	129 (123–137)	127 (117–137)	0.025
Protein (g/kg/d)	3.8 (3.4–4)	3.6 (3.3–3.9)	0.063

### Weight gain before and after start of exogenous digestive enzyme replacement

Daily weight gain per body weight, weight gain for energy and protein intake increased after initiation of exogenous digestive enzyme replacement (Table 3a). Negative deviation from projected weight trajectories decreased, as quantified by weight for postmenstrual age percentiles (Table 3a). The fraction of infants with poor growth (less than 17 g/kg/d) decreased from 46/71 (66%) to 23/71 (32%) (*p* < 0.001).

**Table 3:** **a** Daily weight gain per body weight, weight gain by energy and protein intake, and percentile change before and after initiation of exogenous digestive enzyme replacement (median [interquartile range]), all patients; **b** Subgroup analysis according to type of feeding, as in **a**. **c** Subgroup analysis according to FPE-1 levels, as in **a**.

a			
	Before Initiation	After Initiation	*P*-Value
**Weight gain** (g/kg/d)	14.7 (7.3-19.7)	19.5 (15.7-24.0)	< 0.001
**Weight gain per kcal** (g/kcal)	0.11 (0.06-0.16)	0.15 (0.13-0.19)	< 0.001
**Weight gain per protein** (g/g)	4.0 (2.0-5.9)	5.4 (4.4-6.5)	< 0.001
**Change in weight-for-postmenstrual-age percentile** (/d)	-0.3 (-1.0 - 0.07)	-0.07 (-0.27 - 0.14)	0.005

b			
Raw mother’s own milk (*n*=26)	Before Initiation	After Initiation	*P*-Value
**Weight gain** (g/kg/d)	17.7 (14.2-24.0)	20.4 (15.7-25.1)	0.118
**Weight gain per kcal** (g/kcal)	0.14 (0.11-0.19)	0.17 (0.13-0.20)	0.137
**Weight gain per protein** (g/g)	5.2 (4.0-6.3)	5.4 (4.4-6.5)	0.159
**Change in weight-for-postmenstrual-age percentile** (/d)	0.00 (-1.00 - +0.20)	0.00 (-0.23 - +0.14)	0.191

Pasteurized mother’s own milk (*n*=29)	Before Initiation	After Initiation	*P*-Value
**Weight gain** (g/kg/d)	15.2 (5.5-18.0)	18.2 (14.6-21.5)	0.071
**Weight gain per kcal** (g/kcal)	0.11 (0.05-0.14)	0.14 (0.11-0.16)	0.031
**Weight gain per protein** (g/g)	3.9 (1.7-4.6)	5.1 (3.7-6.1)	0.025
**Change in weight-for-postmenstrual-age percentile** (/d)	-0.33 (-0.86 - +0.04)	-0.15 (-0.53 - +0.14)	0.222

Donor milk (pasteurized) (*n*=9)	Before Initiation	After Initiation	*P*-Value
**Weight gain** (g/kg/d)	9.0 (1.4-13.7)	24.3 (18.5-31.3)	0.011
**Weight gain per kcal** (g/kcal)	0.06 (0.01-0.08)	0.17 (0.13-0.24)	0.011
**Weight gain per protein** (g/g)	2.3 (0.3-3.1)	6.9 (4.9-8.1)	0.008
**Change in weight-for-postmenstrual-age percentile** (/d)	-0.50 (-1.11 - -0.11)	0.0 (-0.12 - +0.12)	0.028

Mixed feeding (*n*=7)	Before Initiation	After Initiation	*P*-Value
**Weight gain** (g/kg/d)	11.3 (0.80-22.7)	20.0 (16.3-24.9)	0.091
**Weight gain per kcal** (g/kcal)	0.09 (0.05-0.18)	0.16 (0.13-0.21)	0.091
**Weight gain per protein** (g/g)	2.9 (1.8-5.8)	6.0 (5.4-8.0)	0.043
**Change in weight-for-postmenstrual-age percentile** (/d)	-0.43 (-1.33 - 0.00)	+0.08 (-0.21 - +0.23)	0.043

c			
FPE-1 ≥ 50µg/g (*n*=36)	Before Initiation	After Initiation	*P*-Value
**Weight gain** (g/kg/d)	14.6 (7.4-20.4)	18.3 (14.8-22.7)	0.010
**Weight gain per kcal** (g/kcal)	0.12 (0.06-0.17)	0.14 (0.12-0.19)	0.007
**Weight gain per protein** (g/g)	4.0 (2.1-6.0)	5.4 (4.0-6.4)	0.006
**Change in weight-for-postmenstrual-age percentile** (/d)	-0.39 (-1.11 - 0.00)	0.00 (-0.21 - +0.23)	0.001

FPE-1 < 50µg/g (*n*=35)	Before Initiation	After Initiation	*P*-Value
**Weight gain** (g/kg/d)	14.7 (7.3-19.4)	19.9 (16.8-24.6)	0.004
**Weight gain per kcal** (g/kcal)	0.12 (0.06-0.12)	0.14 (0.12-0.14)	0.004
**Weight gain per protein** (g/g)	4.0 (2.1-6.0)	5.4 (4.0-6.4)	0.003
**Change in weight-for-postmenstrual-age percentile** (/d)	-0.14 (-0.86 - +0.11)	-0.08 (-0.36 - +0.09)	0.340

### Adverse effects

No adverse effects attributed to the exogenous digestive enzyme replacement were reported, with possible exception of diaper rash.

### Role of confounders

Subgroup analysis according to type of feeding revealed non-significant changes associated with digestive enzyme replacement in infants fed raw mother’s own milk but marked changes in infants fed pasteurized mother’s own milk or donor milk (Table 3b). There were no significant differences in FPE-1 levels in groups defined by feeding type, and effects were similar in infants with FPE-1 levels< or ≥50 µg/g (Table 3c).

Moreover, weight gain both before and after initiation of exogenous digestive enzyme replacement did not differ between infants with or without major intraventricular hemorrhage, no, mild, moderate or severe bronchopulmonary dysplasia, infants who did or did not undergo treatment for patent ductus arteriosus, or infants included at the two different study sites (*p* > 0.1 for each comparison). Weight gain before but not after initiation of exogenous digestive enzyme replacement tended to be lower in infants who later developed severe (grade 3) retinopathy of prematurity, as opposed to infants who had no or less severe retinopathy of prematurity (*p* = 0.057).

## Discussion

In this prospective observational study, administration of exogenous digestive enzyme replacement was associated with significantly improved growth of very low birth weight infants with transient exocrine pancreatic insufficiency. Improved growth was noted for weight gain per kg body weight, as well as weight gain in relation to energy and protein intake. The improved weight gain observed was therefore most likely due to more effective food digestion but not to increased food intake. Negative deviation from growth trajectories was ameliorated but not abrogated by exogenous digestive enzyme replacement.

The results support the notion that exocrine pancreatic insufficiency is a major contributor to postnatal growth faltering in very immature infants, at least those fed pasteurized mother’s own milk or donor milk. Previous retrospective studies have reported a positive association between enteral administration of pancreatic enzymes and improved weight gain in very preterm infants [15, 25]. This is the first prospective study evaluating a commercially available enzyme preparation of microbial origin, with each infant serving as his or her own control. However, there was no control group consisting of infants with low FPE-1 without digestive enzyme replacement, further emphasizing the need to assess this approach in a randomized controlled double-blind interventional trial.

While exogenous digestive enzyme replacement cut the rate of infants with growth failure, defined as daily weight gain of less than 17 g/kg/d [1] from almost 66% to less than 33%, some infants continued to gain weight at a slow pace, despite receiving high amounts of energy and protein. While digestive enzyme replacement appeared to have slowed down loss of weight percentiles, there was no apparent catch-up growth. Of note, the dosages of digestive enzyme administered were chosen based on previous reports, but some infants might have required higher amounts. This study was not designed to assess incremental dosage of digestive enzymes or to gauge the dosages required by the degree of growth failure or fecal elastase-1 concentrations, i.e. clinical and laboratory signs of exocrine pancreatic insufficiency.

In very preterm infants, full digestion of food becomes pivotal once they are on full enteral feeds. FPE-1 was determined at about this time at 3 weeks of live on average, and digestive enzyme replacement started thereafter. However, digestive enzyme replacement could also speed up incremental enteral feeding while the infant is still receiving supplemental intravenous nutrition. Digestive enzyme replacement could also lower the risk milk curd obstruction resulting from poorly digested fortified milk [34] and there was indeed no case of lactobezoar in infants given digestive enzyme replacement.

We used a licensed mixture of lipase, protease and amylase similar in composition to the compounded prescription employed previously [15]. While there is little role for amylase in this setting, protein accretion is critical for the growth of preterm infants [35], and protein but not fat is an essential part of human milk fortification for very preterm infants [36]. The effect of digestive enzyme replacement was mostly restricted to infants receiving pasteurized mother’s own milk or donor milk. Pasteurized milk might be more difficult to digest than native milk, and pasteurization inactivates digestives enzymes present in human milk, notably bile salt-stimulated lipase. In a randomized placebo-controlled trial, addition of recombinant bile salt-stimulated lipase alone to feeding in infants born before 32 weeks’ gestation did not result in a statistically significant improved weight gain with the exception of small for gestational age infants [37]. We would consider both lipase and protease important for digestive enzyme replacement, while their optimal dosages remain to be determined. Future trials are needed to further validate the findings presented here and evaluate potential long-term effects of exogenous enzyme replacement on growth, metabolic development and neurocognitive outcomes.

Initiation of digestive enzyme replacement was triggered by inadequate weight gain and low FPE-1 concentrations determined upon discontinuation of intravenous lines at around 3–4 weeks of age. Earlier measurements would have found more infants with low FPE-1 concentrations while the clinical consequences of exocrine pancreatic insufficiency emerge once an infant is on full enteral feeds.

As a further limitation, fortification of breast milk feeds was done based on average protein and fat concentrations and kept constant [38], rather than being adjusted in response to infant blood urea concentrations or targeted to the breast milk macronutrient content measured with a milk analyzer [39]. Fat and protein contents of pumped breast milk from mothers of very preterm infants display considerable within-subjects and between-subjects variation [40]. In our calculations, we made no correction for the longitudinal decline of protein content of human milk produced by mothers of preterm infants [41] which might have led to an underestimation of the effect of digestive enzyme replacement on weight gain per g protein fed. Some infants may need more macronutrients or benefit from additional sodium supplementation [42].

The results of this prospective observational study suggest that enteral administration of exogenous digestive enzymes may be a promising and safe approach to support the growth of very preterm infants with transient exocrine pancreatic insufficiency. This intervention could help to reduce the postnatal growth deficit in this vulnerable population of patients and may thereby offer long-term health benefits. While this before—after comparison within the same group demonstrates an association between administration of exogenous digestive enzymes with improved growth of very low birth weight infants, the design of the study cannot prove causality. However, the data of this study may encourage and help to design a double-blind randomized controlled trial to further evaluate the merits of digestive enzyme replacement in very preterm infants.

## Data Availability

Data of this study are available upon reasonable request after granted approval of the Ethikkommission der Charité and the institutional data protection officer.
